# O-43 - A REGIONAL INCREASE IN CLADE IIB MPOX IN THE NORTH WEST ENGLAND LINKED TO SEX ON PREMISES VENUES

**DOI:** 10.1093/pubmed/fdag046.038

**Published:** 2026-07-09

**Authors:** Mr Reece Jarratt, Thomas Inns, Maria SuauSans, Jon Dunn, Sarah JohnsonGriffiths, Evdokia Dardamissis

**Affiliations:** UK Health Security Agency; UK Health Security Agency; UK Health Security Agency; UK Health Security Agency; UK Health Security Agency; UK Health Security Agency

## Abstract

**Background:**

Following the global re-emergence of mpox, clade IIb transmission in England has been concentrated in London^1^. Community transmission has been primarily in sexual networks of gay, bisexual, and other men who have sex with men (GBMSM)^2^. In late 2025, an unusual increase in clade IIb mpox cases was detected across the North West, representing a rare cluster outside London and prompting enhanced surveillance and incident management.

**Objectives:**

To characterise the epidemiology and transmission settings associated with an increase in clade IIb mpox cases in the North West of England.

**Methods:**

Laboratory-confirmed clade IIb mpox or orthopox (highly probable mpox clade IIb) cases identified, with a specimen dates between 01 October 2025 and 09 April 2026, among residents of the North West were included. Demographic and exposure data were obtained through enhanced case interviews. Spatiotemporal trends, demographic characteristics, and reported exposure settings were described using R^3^.

**Results:**

A total of 72 cases were reported, with cases peaking in February 2026 (n=35,49%). The majority of cases were male (n=71,99%), aged 30–39 years (n=31,43%), and residents across Greater Manchester (n=49,68%). Among male interview respondents (n=43), 34(79%) self-identified as GBMSM.

Case interviews identified an sex-on-premises venue as an early potential transmission setting, prompting urgent public health action, including a site visit and targeted risk communication. Increased awareness among case interviewers subsequently led to the identification of two additional sex-on-premises venues associated with transmission to varying degrees.

**Conclusions:**

This represents one of the largest clade IIb mpox clusters identified outside London to date. The findings highlight the importance of enhanced surveillance and interviewer awareness in identifying transmission settings, and the effectiveness of rapid, co-ordinated multi-agency public health action.

References

1. UK Health Security Agency. *Mpox outbreak: epidemiological overview, 9 April 2026* [Internet]. London: UK Health Security Agency; 2026 Apr 9 [Accessed 09 April 2026]. Available from: https://www.gov.uk/government/publications/monkeypox-outbreak-epidemiological-overview/mpox-outbreak-epidemiological-overview-9-april-2026

2. Vivancos R, Anderson C, Blomquist P, *et al.* Community transmission of monkeypox in the United Kingdom, April to May 2022. Euro Surveillance. 2022;27(23):220609c. doi:10.2807/1560-7917.ES.2022.27.23.220609c

3. R Core Team. R: a language and environment for statistical computing. Vienna: R Foundation for Statistical Computing; 2026. Available from: https://www.r-project.org/

